# The initial infectious dose of SARS-CoV-2 and the severity of the disease: possible impact on the incubation period

**DOI:** 10.2217/fvl-2020-0330

**Published:** 2021-04-14

**Authors:** Hamid Tayebi Khosroshahi, Alireza Mardomi

**Affiliations:** 1 ^1^Kidney Research Center, Tabriz University of Medical Sciences, Tabriz, Iran; 2 ^2^Immunogenetics Research Center, Mazandaran University of Medical Sciences, Sari, Iran

**Keywords:** cytokine storm, incubation period, initial infectious dose, SARS-CoV-2, viral load

## Abstract

Clinical presentations of COVID-19 exhibit a relative variation that might have roots in various circumstances. The initial infectious dose is a decisive factor in determining the fate of some viral and bacterial infections. Regarding the importance of inflammation and immunopathogenesis in the progression of COVID-19, the initial infectious dose of severe acute respiratory syndrome coronavirus 2 might have an impact on the initial inflammation and therefore the incubation period of the disease. A quick progression to severe disease might decrease the chance for successful therapy. Therefore, more release of danger-associated molecular patterns and more cytokine responses are expectable in the case of higher infectious doses. Herein we discuss the impact of initial infectious dose in the pathogenesis of similar infections such as MERS and influenza.

The outbreak of COVID-19 has been turned into a serious pandemic health problem and caused considerable mortality all around the globe. Despite extensive researches on the physiopathology of this infection, there are still numerous gaps in our knowledge on the virulence of severe acute respiratory syndrome coronavirus 2 (SARS-CoV-2). One of the interesting issues regarding the COVID-19 is a relative variation in clinical presentations of infected individuals ranging from asymptomatic and mild presentations to acute respiratory distress syndrome (ARDS) and multi-organ damage [1]. The causative mechanisms for the observed variations in the presentations and the severity of the disease are generally the host- and virus-associated factors. The host factors determining the susceptibility to COVID-19 could be summarized as HLA haplotypes [2], the status of innate immunity [3], T helper 1/T helper 2 (Th1/Th2) balance [4], cytokine signature [5] and the expression level and polymorphisms of *ACE2* [6,7], and others. The virus-associated factors could be explained as various mutations affecting the virulence of SARS-CoV-2 [8].

## Pathogenesis & immunology of COVID-19

By the immunologic prospect, SARS-CoV-2 infection could be divided into the incubation and severe stages. While during the incubation period, effective immune responses are required for limiting the infection, immune responses are generally deteriorating in severe stages. However, efficient and specific T- and B-cell adaptive responses can impede the disease progression to severe forms. As indicated above, host factors such as HLA background, plays a critical role in determination of the fate of the infection. It has been shown that some HLA haplotypes are capable of presenting the SARS-CoV-2 antigens more potently [9]. When an HLA haplotype fails to present the immunodominant antigens of SARS-CoV-2, adaptive responses will be absent, allowing the expansion of the thriving virus eventuating in the disease progression severe stage. There are distinct therapeutic strategies for coping with the infection in incubation period and severe stages. Approaches for reinforcing the immune system such as convalescent plasma therapy and IFN-α therapy could be advantageous at initial stages. These therapies might reinforce the host in establishment of infection-limiting responses. By the spread of the virus, the ACE2 expressing tissues such as gastrointestinal and renal tissues could get infected resulting in the disease stage shift to severe stages. The involvement of these tissues is accompanied by a heavy release of danger-associated molecular patterns (DAMPs). These events initiate a cascade of inflammatory events culminating in severe lung infiltration with inflammatory cells. During these steps, immunomodulation is beneficial to harness the detrimental inflammatory responses [9,10]. COVID-19 is accompanied by lymphopenia, atrophy of lymphoid tissues, and the infiltrations of neutrophils and monocytes into the tissues involved [11]. Cytokine release storm is one of the characteristics of the inflammatory disease during the progress of COVID-19. Activated monocytes and T cells are the main sources of inflammatory cytokine release in response to the disseminated inflammatory mediators. A high magnitude of pro-inflammatory cytokines could have detrimental impacts on various organs. There are other pathologic conditions associated with cytokine release storm such as rheumatologic diseases, infections and immunotherapies [12]. In the case of infection, a localized cytokine response can eventuate in a systemic cytokine release storm. During severe SARS-CoV-2 infection, heavy pro-inflammatory cell infiltration and cytokine release storm are the main explanations for the lung damage and ARDS that is the main mortality cause in COVID-19 [9,10]. While in other cytokine release storm situations, the hepatosplenomegaly is evident [12], COVID-19 causes the atrophy of lymphoid organs. The sustained cytokine release storm causes the huge death of lymphocytes causing their counts to experience a sharp drop in peripheral and lymphoid organs [11]. The sustained inflammation not only exerts detrimental effects on organs such as kidney and liver [12] but also contributes to alveoli endothelial and epithelial cell activation. Then, these cells acquire a proinflammatory phenotype by upregulation of cell adhesion molecules, pattern recognition receptors such as TLRs, NLRs, and other innate immunity receptors [9]. One of the critical outcomes of cytokine release storm is the upregulation of *HAS-2*. The overexpression and activity of *HAS-2* causes the heavy accumulation of hyaluronan in alveolar cavities. Hyaluronan disturbs the alveolar function by a jelly within alveoli by the means of water absorption [10,13]. Thus, the fatal ARDS accompanied by COVID-19 is an immunopathogenic event. This explains the effectiveness of immunosuppressants and immunomodulators in the treatment of severe cases of COVID-19 [11].

## The infective dose & COVID-19

**Figure 1.**
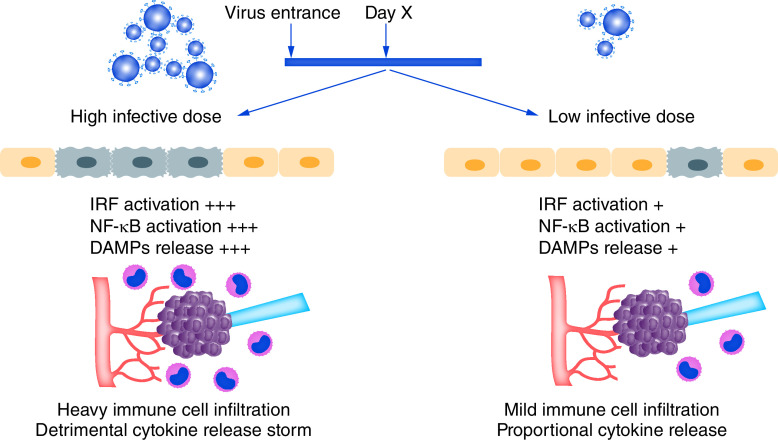
Differential consequences of infections with high and low colony-forming units of SARS-CoV-2 in a constant time post virus entry. Hypothetically, a high magnitude of virus causes a robust activation of innate immunity and inflammation rather than low colony-forming units of the virus at a similar time post virus entrance.

We hypothesize here that the colony-forming units of SARS-CoV-2 in the entry time might be a decisive factor, affecting the disease progression to severe step (Figure 1). The more infective dose of virus, the more innate immune response. Therefore, increasing the probability of cytokine storm and cytokine-associated organ damage in a constant period post the virus entrance. As an example, in tuberculosis, the dose of the infectious agent is critically important in determining the fate of the disease. So that, a low dose of infective bacteria increases the likelihood of latent tuberculosis infection rather than the acute forms of the disease. High doses of bacteria might result in elevated involvement of innate immunity and therefore better stimulation of adaptive responses. It has been shown that a sustained Th1 response accompanied by pro-inflammatory cytokines eventuates in persistent inflammation and granuloma formation. The good prognosis of tuberculosis has been associated with low IFN-γ and high TGF-β responses [14]. Therefore, granuloma-associated lung damage is an immunopathogenic situation. The mentioned condition is comparable to severe stages of COVID-19 infection. It seems that upon the entry of high colony-forming units of SARS-CoV-2, higher numbers of target cells could be infected. Accordingly, more release of DAMPs and more interferon responses are expectable. This pro-inflammatory situation leads to exacerbation of the inflammatory condition by causing a heavy immune cell infiltration and cytokine release storm. The high replication rate of the pathogen and the inability of the immune system in virus clearance further triggers the detrimental immunopathogenic pathways. In addition to DAMPs, TLR-7- and NOD2-mediated inflammation caused by virus components reinforce the progressive inflammatory condition even more [15–17]. Hence, it seems that the higher the count of initially infected cells, the higher the inflammatory mediators produced. This phenomenon might cause the incubation period of the infection to be shortened and the disease to be converted to the severe stage more quickly. Although immune cells such as neutrophils, macrophages and subsequently T cells accumulate in the infected tissues [10], a high replication rate of the virus along with an infection-induced exhaustion phenotype renders the immune system unable to establish a functional response for efficient combating the virus-infected cells [18,19]. High activation of immune cells within the infected tissue results in consequences such as lymphocyte activation-induced cell death and pathologic activation of alveolar endothelial and epithelial cells [10]. Therefore, time is a decisive factor in the progression of COVID-19. So that, if the initial infective dose of SARS-CoV-2 be so high to infect plenty of target cells, the innate and adaptive immune systems might fail to overcome the virus replication in the incubation period. A too-short incubation period might decrease the chance of response to therapy, as well. In similar conditions such as in SARS-CoV-1 and middle east respiratory syndrome (MERS), the initial infectious dose is a determining factor for disease progression to the severe respiratory syndrome [20–22]. It has been shown that some evolved MERS-coronaviruses can cause respiratory involvement with lower doses compared with the other MERS viruses [23]. Moreover, in influenza infection, the viral load is positively correlated to disease severity [24]. However, the only report examining the association between the load of SARS-CoV-2 and the severity of the disease, reports no significant correlation. This study focuses on the viral load in the disease course rather than the initial infecting dose [25]. However, there are some indications for the involvement of the initial infectious dose in the pathogenesis of COVID-19 [26]. It seems that this hypothesis could be confidently tested in animal models of COVID-19. In the case of humans, the main group proposed to be possibly infected with higher doses of SARS-CoV-2 are the healthcare workers. In Italy, the mortality rate among the SARS-CoV-2 positive healthcare workers was 0.014 while the mortality rate of other COVID-19 sufferers was 0.143 [27]. On the other hand, the mortality rate of Iranian healthcare workers infected with SARS-CoV-2 was higher than the other COVID-19 cases (0.135:0.077). However, these statistics might be not reliable regarding the different screening policies in various countries and considerable numbers of asymptomatic COVID-19 cases. Besides, the countries that experienced the outbreak earlier might have provided fewer precautions for the at-risk personnel in the earlier steps.

## Future perspective

Altogether, herein we exhibited some logics for probable participation of SARS-CoV-2 infective dose in the progression of COVID-19. Higher colony-forming units of the virus might shorten the incubation period of the disease and accelerate the establishment of severe complications of COVID-19. Nevertheless, the exact role of the virus infective dose in the pathogenesis of SARS-CoV-2 remains to be defined. Specific studies in this case will increase our knowledge about the possible correlation between infective dose and COVID-19 progression. This is specifically important in protecting high-risk individuals such as social caretakers and healthcare workers in occupational encounters.

Since determining the initial infectious dose in human studies is a complex procedure, humanized mouse models and COVID-19-specific models might provide a platform for precise evaluations on the associations between the infectious dose of SARS-CoV-2 and the severity of the infection. Thus, the kinetics of viral infection and inflammatory responses could be easily tracked in such animal models.

**Executive summary**

**Pathogenesis & immunology of COVID-19**

- COVID-19 represents kinds of heterogenicity in representations among various patients that the majority of them were attributed host factors.

**The infective dose & COVID-19**

- Similar clinical conditions such as Middle East respiratory syndrome, severe acute respiratory syndrome coronavirus-1 and influenza exhibit correlation between the initial infective dose and the severity of the complications and disease progression.
- It has been shown that some healthcare workers such as tracheal tube implementation staff that might be in contact with higher colony-forming units of severe acute respiratory syndrome coronavirus-2 exhibit more severe forms of COVID-19.
- The initial infective dose of SARS-CoV-2 might be a determining factor in the outcome of COVID-19 by affecting the incubation period of the infection.
